# Antiproliferative Action of Methanolic Petiole Extract of Eichhornia Crassipes on Human Prostate Adenocarcinoma Cell Line: An In Vitro Study

**DOI:** 10.7759/cureus.32616

**Published:** 2022-12-16

**Authors:** Noufal K P, Rajesh B, Sujith S Nair

**Affiliations:** 1 Department of Anatomy, Bharath Institute of Higher Education and Research, Chennai, IND; 2 Department of Anatomy, Sri Lakshmi Narayana Institute of Medical Sciences, Puducherry, IND; 3 Department of Pharmaceutics, Crescent College of Pharmaceutical Sciences, Pazhayangadi, IND

**Keywords:** mtt assay, prostate cancer, probit analysis, pc3 cell line, eichhornia crassipes

## Abstract

Background: An increasing number of people are turning to herbal medicines in their search for innovative pharmaceuticals since they are effective treatments for a wide variety of conditions and traditional herbs are rich in bioactive chemicals. In this study, we looked at whether or not a petiole extract of Eichhornia crassipes preserved in methanol inhibited the proliferation of prostate cancer (PC3) cell lines.

Materials and methods: Lakes in Ezhikkara, Ernakulum, Kerala, were the source of E. crassipes. Soxhlet extraction was used to create the extract. 3-[4,5-dimethylthiazol-2-yl]2,5-diphenyltetrazolium bromide (MTT) assay was used to determine the cell viability of methanolic petiole extract at various concentrations. Mean and standard deviation was used to determine absorbance scores. Utilizing probit analysis, we determined the IC50 value. The descriptive statistics to measure the percent of viable cells along with the regression equation were calculated using SPSS.

Results: It has been shown that the methanol extract significantly impacted PC3 cell lines' capacity to survive. It was also determined that increasing the medication concentration resulted in a decrease in cell viability. The percentage of living cells was measured after being exposed to methanol extracts at concentrations of 12.5 μg/ml, 25 μg/ml, 50 μg/ml, 100 μg/ml, and 200 μg/ml, and found to be 95.13, 85.88, 76.12, 64.33, and 53.62 percent, respectively. With IC50 values of 199.488 g/ml, it was shown that methanolic petiole extracts of E. crassipes are cytotoxic. Using probit analysis, we determined that the regression equation is y = -0.2051x + 90.915, with an R2 value of 0.893.

Conclusion: As a result of its chemotherapeutic properties, the E. crassipes petiole extract has the potential to be employed in therapeutic applications, with the ultimate goal of bettering prostate cancer management practices and clinical results by drastically lowering cell viability. The study's results may pave the way for fresh chemotherapeutic approaches to be developed for the treatment of androgen-independent prostate cancer.

## Introduction

When it comes to male mortality, prostate cancer (PC) is both the most well-known malignant development and the second leading cause of death. Most PC are adenocarcinomas [1], which can be identified by the lack of basal lamina and the unchecked growth of malignant neoplastic cells displaying luminal separation through characteristics like organ arrangement and the confirmation of androgen receptor (AR) and prostate-specific antigen (PSA) articulation. As a result, PC has been extensively studied for a long time. The Lymph Node Carcinoma of the Prostate (LNCaP) cell line, which originates from a lymph hub metastasis, and the PC3 cell line, which originates from a bone metastasis, are the two most often used cell lines for studying the various subtypes of PC. Multiple studies show that LNCaP cells, which express AR and PSA, are the relatively asymptomatic androgen-dependent variant over a longer period of time. Conversely, PC3 cells are an aggressive form of androgen-independent PC that lacks AR and PSA [1].

There has been an uptick in the use of medicinal herbs in the search for innovative medications since traditional herbs are such rich sources of natural bioactive chemicals that have been used to relieve and cure a wide range of diseases. The realization that phytochemicals have the ability to treat human illnesses is explicitly established as the basis for the development of new prescription drugs. The leaves, stems, flowers, petioles, and seeds are all found to contain bioactive substances. Herbal treatments are increasingly being used in cancer care, and these treatments point to a wealth of novel and highly bioavailable chemicals for the development of new chemotherapeutics, with the added benefit of exhibiting desirable and relatively lesser toxic effects compared to standard cytotoxic drugs [2]. According to their structure and function, phytoconstituents and secondary metabolites are separated into a number of distinct categories. Flavonoids are a kind of polyphenol that is often found in plant-based foods. Composed of two benzene rings joined by a heterocyclic pyran ring, they are referred to as a benzo-pyrone configuration. Antitumor activity, hepatoprotective, antioxidant, cytoprotective, immune-mediator, antidiabetic, and antibacterial is only a few of their well-known therapeutic possibilities [3].

In the early stages of PC, androgens promote cell proliferation, which can be suppressed by a number of therapeutic approaches that either reduce circulating androgens or aim to block ARs using antagonists. However, cells in the advanced stage of PC are generally resistant to the androgen effect and continue to proliferate uncontrollably. Despite the widespread use of antiandrogenic therapy, a sizable percentage of PCs still enter a refractory phase. More targeted and effective anticancer and chemoprotective medicines are needed [4]. Several chemotherapeutic drugs have recently been licensed for clinical application in an attempt to manage PC. Scientific investigation has revealed that chemically manufactured medicinal substances are harmful, but phytochemicals like polyphenols are safe. By contributing electrons to reactive oxygen species, these chemicals efficiently neutralize them. Antioxidant, anticarcinogenic, and antiviral capabilities are only some of the many benefits associated with phytophenolic substances [5]. Grape seed, catechins, lycopene, isoflavones, and extracts of herbal tea have all been shown to reduce the development of PC cells [4].

Floating waterweed Eichhornia crassipes (Pontederiaceae) is often known by its common name Water Hyacinth [6,7]. Crude whole-plant methanol extracts were very effective in in vitro studies [8,9] using Michigan Cancer Foundation-7 (MCF-7), Henrietta Lacks (HeLa), European Collection of Authenticated Cell Cultures (ECACC), and HepG2 cell lines. The aqueous leaf extract of E. crassipes was able to inhibit the NCI-H322 cell line while protecting the T47D cell line from harm [6,10]. One study shows that PC3 (human prostate cancer) cell lines are resistant to E. crassipes extract [6], but otherwise, there has been very little investigation into the antiproliferative effect of E. crassipes. Because of this, we set out to see if we could stop the growth of the PC3 cell line using an extract from the petiole of the E. crassipes plant.

## Materials and methods

Collection and identification of E. crassipes

E. crassipes were caught in the seas off of Ezhikkara, Ernakulum, Kerala. The validity of the plant was confirmed by botanists from the University of Calicut's Department of Botany. Lagoons and verdant fields surround the village of Ezhikkara in the Paravur Panchayat. The coordinates for its location are 10° 8' 25.7856 North and 76° 13' 49.8792 East. Government officials in the area have begun spreading the word about the harm E. crassipes causes to the aquaculture industry and have begun taking steps to eradicate the pest from local water supplies. Later on, an attempt was made to examine its potential anticancer effects on malignant cells in vitro.

Methanol extract preparation and MTT assay

The entire plant was dug up from the water, and its leaves and stems were washed many times with running water. Before being air-dried or lyophilized, it was sanitized using sterile water. The plant parts, including the leaves, petioles, and rhizomes, were extracted and then finely chopped. Based on the phytochemistry analysis from the prior work [11], methanol extracts of petioles exhibited much higher concentrations of chemotherapeutic components. In this study, the petioles were chosen for the 3-[4,5-dimethylthiazol-2-yl]2,5-diphenyltetrazolium bromide (MTT) test, which measures the quantity of inhibitory action at different doses. The petioles were then fractionated in a heated mantle with methanol using the soxhlet extraction technique after being crushed into uniform particles. Not long after the fractions were extracted, the solvent was recovered by distillation in a rotary evaporator at 40 °C. To preserve them, the extracts were dried under a vacuum and stored in the fridge.

The sample size was determined based on the previous similar study conducted by Abel and Baird [12] on cytotoxic activity toward PC. The sample size was 5000 cells/well which was a lab study where in vitro quantitative variables of the percent of viable cells were tested. The study was an observational study conducted in a laboratory setting (in vitro). The variable analyzed was the percentage of cell viability of the PC3 cell line following the exposure of various concentrations of the petiole extract of E. crassipes.

Evaluation of antiproliferative activity by MTT assay

Cell Lines, Culture Media, Preservation, and Maintenance

The PC3 cell line (Cat. No. 21127) was originally derived from bone metastases in patients with stage IV PC and is routinely cultured in Ham's F12K (Kaighn's modification of Ham's F12 medium) media containing 10% fetal bovine serum (FBS). Liquid nitrogen was used to flash-freeze the cell lines. The lower sodium bicarbonate concentration is meant for use with air containing 5% carbon dioxide.

Cell Seeding in 96-well Plates

Trypsinization, the process by which confluent cells in a tissue cultured (TC) flask are harvested for further transplantation or seeding in 96-well plates, was used on an estimated 80% to 90% of adherent cultivated cells. Phosphate-buffered saline containing 0.025% trypsin and 0.01% ethylenediaminetetraacetic acid was used to treat a single layer of cultured cells on a TC flask. To achieve a concentration of 5x10^3^ cells per well in 100 l, the cell culture medium was diluted with trypsinized cells. After seeding the cells in 96-well plates, they were kept in the incubator for three to four days.

Sample Preparation and Treatment

Cultures of the evaluation samples were made in Ham's F12K medium at a concentration of 100 mg/ml, and the resulting solution was filtered using a 0.2 m Millipore syringe filter. Next, 12.5 μg, 25 μg, 50 μg, 100 μg, and 200 μg dilutions of the materials were seeded onto 96-well plates containing cell cultures. Controls consisted of two empty wells on each plate. All studies were performed in triplicate, and the means were calculated to minimize the possibility of error. Forty-eight hours of incubation followed after the plates were treated with the test specimens.

In Vitro Screening of Cytotoxicity

Using an MTT experiment, we calculated the percentage of PC-3 cell growth inhibition. Approximately 105 PC3 cells were planted in a 96-well plate and incubated for 24 hours at 37 °C with 5% CO_2_. PC-3 cells were treated with a serial dilution of petiole extract the next day and then incubated for 48 hours before being exposed to 20 μl of 5 mg/mL of MTT solution in the dark. Then, it was wrapped in aluminum foil and hidden for four hours. The purple formazan crystals were dissolved by adding 100 μl of dimethyl sulfoxide to each well after the medium was removed. In order to decipher the plate's contents at the 570 nm absorption wavelength, a microplate reader will be employed. To determine the IC50 of petiole extract's antiproliferative effect on PC3 cells, the experiment was performed three times.

Direct Microscopic Observation

A tissue culture microscope equipped with an upset stage contrast was used to take pictures of the treatment and control wells at regular intervals for 48 hours. Cytotoxic effects were thought to manifest themselves in a number of ways, including cytoplasmic rounding, chromatin condensation, granulation, and vacuolization. When the incubation period was through, the wells were drained dry and their contents discarded. The 570 nm absorption spectrum optical density was measured and compared to the control using a microplate reader. Using the following formula, we were able to determine the percentage of inhibition of cell growth:

“Percentage of cell viability = (Average absorbance of treated/Average absorbance of control) x 100.”

Statistical Analysis

Statistical Product and Service Solutions (SPSS) (IBM SPSS Statistics for Windows, Version 26.0, Armonk, NY) was used for all statistical analysis. The percentage of cell growth inhibition was determined using the absorbance measurements (shown as Mean+SD). Utilizing the slope of the regression equation, y = mx + c, we were able to determine the IC50 using the probit model.

## Results

The anticancer properties of a methanol extract of the petiole of E. crassipes were examined using the PC3 cell line (Table 1).

**Table 1:** Probit analysis of the methanolic petiole extract of Eichhornia crassipes against PC3 cell lines µg/ml - microgram per milliliter PC3 - prostate cancer cell line

			Drug concentration unit: µg/ml (cell line: PC3)				
Parameter	Blank	Untreated	12.5	25	50	100	200
Abs reading 1	0.042	1.359	1.327	1.186	1.045	0.896	0.758
Abs reading 2	0.038	1.381	1.312	1.195	1.083	0.902	0.739
Abs reading 3	0.031	1.416	1.320	1.204	1.062	0.915	0.783
Mean abs	0.037	1.385	1.320	1.195	1.063	0.904	0.760
Mean abs (Sample-Blank)		1.348	1.283	1.158	1.026	0.867	0.723
Standard deviation		0.029	0.008	0.009	0.019	0.010	0.022
Standard error		0.017	0.004	0.005	0.011	0.006	0.013
Cell Viability %		100	95.13	85.88	76.12	64.33	53.62

**Figure 1:**
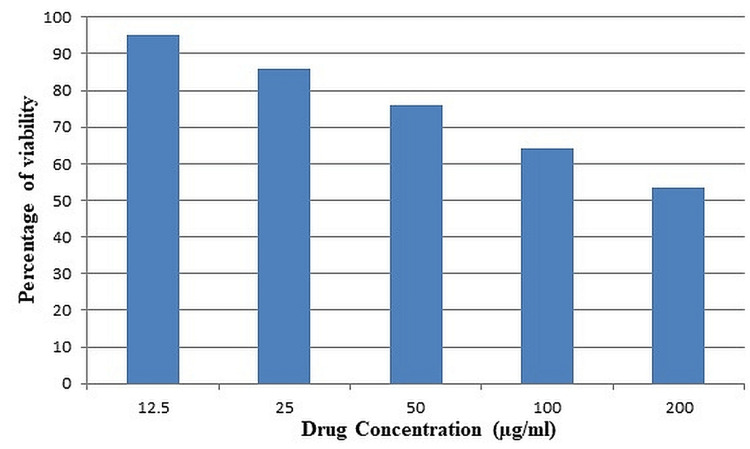
Cytotoxic action of methanolic petiole extract of Eichhornia crassipes against PC3 cell lines PC3 - prostate cancer cell line

The results showed that methanolic extracts lowered the viability of tumor cell lines. At 12.5 μg/ml, 25 μg/ml, 50 μg/ml, 100 μg/ml, and 200 μg/ml, the percentages of viability were 95.13, 85.88, 76.12, 64, and 53. The percentage of live cells decreased with increasing drug concentration (Figure 1).

**Figure 2:**
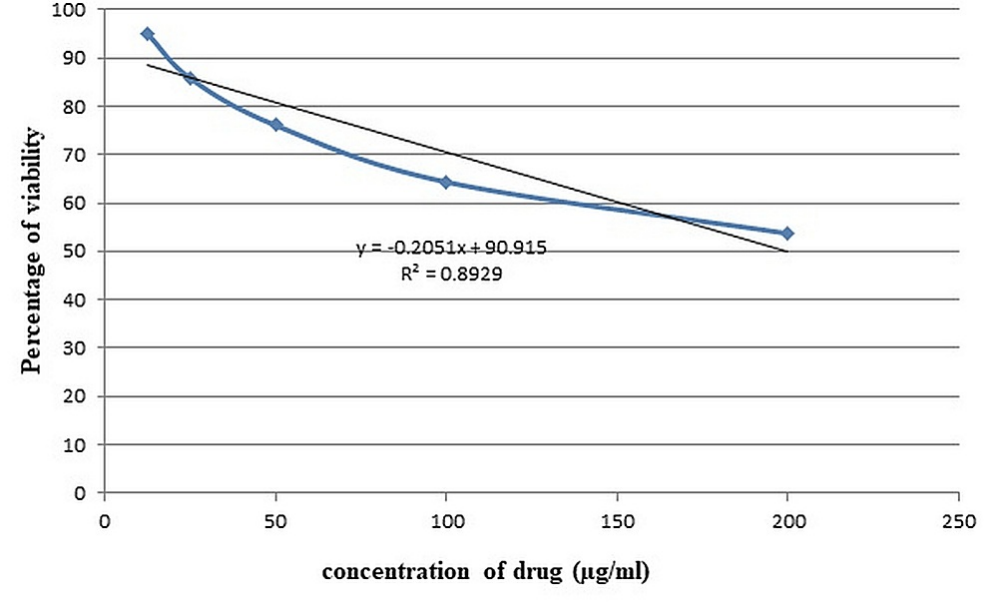
Dose-response curve of methanolic petiole extract of Eichhornia crassipes against PC3 cell lines PC3 - prostate cancer cell line

In Figure 2, we can see how the concentration of E. crassipes extract affects the inhibition of PC3 cell growth.

**Figure 3:**
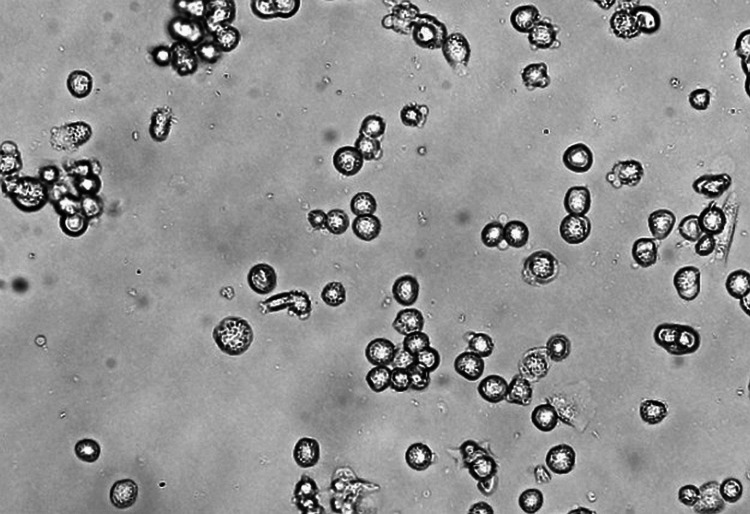
Anticancer activity of methanolic extract of Eichhornia crassipes against PC3 cell line PC - prostate cancer cell line

Depending on the concentration, the extract significantly slowed the growth and proliferation of test cell lines. Using probit analysis, we derived a regression equation (y = -0.2051x + 90.915) with a coefficient of determination (R2) of 0.893. A comparison of PC3 cells treated with 200 μg/ml of the extract (a) and untreated cells (b) demonstrates the extract's antiproliferative action. Viable PC3 cells were reduced in number after being treated with varying concentrations of an extract of the methanolic petiole of the plant, Epilobium crassipes. The IC50 values for E. crassipes methanolic extracts against the PC3 cell line proliferation were determined to be 199.488 g/ml of test sample concentration, indicating the extracts' efficacy (Figure 3).

## Discussion

The PC-3 cell line is the gold standard for studying PC. It is used to mimic PC that does not respond to androgens. This cell line is more aggressive than previous PC models [13]. Thus, the purpose of this study is to examine whether or not PC3 cells can be inhibited from multiplying by a substance found in the petiole of E. crassipes. Inhibiting cell growth and inducing apoptosis through androgen deprivation treatment can help alleviate the androgen-dependent phase of PC, leading to the involution of the prostate. Androgen independence is a fatal stage to which many patients inevitably advance, but it is not treatable at this time. Therefore, in-depth studies are required to learn about the androgen-independent disorder and to find the most efficient PC treatments [13].

Normal treatments target rapidly multiplying abnormal cells, leading to complete tumor regression. However, the fraction of cancer cells that did survive replicates and triggers multi-lineage differentiation in order to reassemble the tumor. The recurrent tumor is a more dangerous and resistant form of the original that changes into a more invasive form over time [14,15]. Once ignored, Eichhornia has recently received a lot of attention due to its usefulness in many other areas [16]. In recent years, scientists have discovered that phytochemicals isolated from different plant components serve a wide variety of purposes [17]. Results from pharmacological and biochemical studies on plant extracts in solvents such as methanol, water, chloroform, and hexane were found to be promising in the published literature. Anti-inflammatory, anti-fungal, anti-aging, anticarcinogenic, hepatoprotective, insecticidal, and larvicidal activities have also been shown for E. crassipes extracts [18-20]. Due to their ability to protect cells from oxidative stress, flavonoids have a significant anticancer potential [21-24]. Flavonoids are powerful water-soluble antioxidants and free radical eliminators. The development of safe, efficient chemotherapeutic medicines that are gentle on host tissues and have potent antioxidant capabilities has traditionally relied heavily on naturally occurring chemicals [25].

A separate prognostic signal in PC patients was found to be an elevated expression of the nuclear factor-kappa B (NF-B)/p65 protein in clinical studies. PC samples that had recurred after radical prostatectomy had high levels of NF-B in their nuclear fraction. Because of this, blocking the NF-B signal transduction pathway is crucial in the fight against PC [13]. The NCl-H322 cell line, T47D cell line, B16F10, hepatocellular carcinoma cells, and HeLa cells have all been shown to be susceptible to the aqueous extract's anticancer effects in laboratory experiments [8,9,26,27]. While our results show that PC3 cell lines are sensitive to aqueous leaf extract, Kumar et al. [6] found the opposite to be true. This is because the physiochemical components of aquatic botanicals and weeds vary greatly according to factors such as the genetic background of the plant, seasonal changes, and geographical location [18]. But there seems to be very little written about how the water hyacinth petiole extract affects PC3. Since phytochemicals derived from the plant have disease-preventive qualities, more research incorporating extracts from diverse sections of water hyacinth may increase the application and value of E. crassipes.

## Conclusions

In order to enhance PC management and prognosis, the E. crassipes petiole extract shows promise as an anticancer treatment because of its capacity to drastically reduce cell viability. New chemotherapeutic techniques for the treatment of androgen-independent PC may be developed on the basis of the study's results.
